# Amentoflavone Mitigates Cyclophosphamide-Induced Pulmonary Toxicity: Involvement of -SIRT-1/Nrf2/Keap1 Axis, JAK-2/STAT-3 Signaling, and Apoptosis

**DOI:** 10.3390/medicina59122119

**Published:** 2023-12-04

**Authors:** Mohamed F. Balaha, Ahmed A. Alamer, Rana M. Aldossari, Alhussain H. Aodah, Azza I. Helal, Ahmed M. Kabel

**Affiliations:** 1 Clinical Pharmacy Department, College of Pharmacy, Prince Sattam bin Abdulaziz University, Al-Kharj 11942, Saudi Arabia; 2 Pharmacology Department, Faculty of Medicine, Tanta University, El-Gish Street, Tanta 31527, Egypt; 3 Department of Pharmacology and Toxicology, College of Pharmacy, Prince Sattam Bin Abdulaziz University, Al-Kharj 11942, Saudi Arabia; 4 Department of Pharmaceutics, College of Pharmacy, Prince Sattam Bin Abdulaziz University, Al-Kharj 11942, Saudi Arabia; 5 Department of Histology and Cell Biology, Faculty of Medicine, Kafrelsheikh University, Kafrelsheikh 33516, Egypt; 6 National Committee of Drugs, Academy of Scientific Research and Technology (ASRT), Ministry of Higher Education, Cairo 11694, Egypt

**Keywords:** amentoflavone, cyclophosphamide, pulmonary toxicity, sirtuin-1, JAK2/STAT3 pathway, apoptosis, rats

## Abstract

*Background and objectives:* Cyclophosphamide (CPA) is an alkylating agent that is used for the management of various types of malignancies and as an immunosuppressive agent for the treatment of immunological disorders. However, its use is limited by its potential to cause a wide range of pulmonary toxicities. Amentoflavone (AMV) is a flavonoid that had proven efficacy in the treatment of disease states in which oxidative stress, inflammation, and apoptosis may play a pathophysiologic role. This study investigated the potential ameliorative effects of the different doses of AMV on CPA-induced pulmonary toxicity, with special emphasis on its antioxidant, anti-inflammatory, and apoptosis-modulating effects. *Materials and methods:* In a rat model of CPA-induced pulmonary toxicity, the effect of AMV at two dose levels (50 mg/kg/day and 100 mg/kg/day) was investigated. The total and differential leucocytic counts, lactate dehydrogenase activity, and levels of pro-inflammatory cytokines in the bronchoalveolar lavage fluid were estimated. Also, the levels of oxidative stress parameters, sirtuin-1, Keap1, Nrf2, JAK2, STAT3, hydroxyproline, matrix metalloproteinases 3 and 9, autophagy markers, and the cleaved caspase 3 were assessed in the pulmonary tissues. In addition, the histopathological and electron microscopic changes in the pulmonary tissues were evaluated. *Results:* AMV dose-dependently ameliorated the pulmonary toxicities induced by CPA via modulation of the SIRT-1/Nrf2/Keap1 axis, mitigation of the inflammatory and fibrotic events, impaction of JAK-2/STAT-3 axis, and modulation of the autophagic and apoptotic signals. *Conclusions:* AMV may open new horizons towards the mitigation of the pulmonary toxicities induced by CPA.

## 1. Introduction

Cyclophosphamide (CPA) is one of the alkylating agents that is used on a wide scale for the treatment of breast, ovarian, and hematologic malignancies [1]. In addition, it exhibits potent immunosuppressive properties that are clinically applied for the management of several pathological conditions characterized by an immunological nature [2]. However, pulmonary toxicity induced by CPA may represent a serious obstacle to the beneficial use of this chemotherapeutic agent [3]. The mechanisms of this toxicity may include perturbations of the pro-oxidant/antioxidant balance, augmentation of the inflammatory pathways, and modulation of the apoptotic signals in the pulmonary tissues [4]. Therefore, targeting these potential mechanisms may contribute to the mitigation of the deleterious effects of CPA on the pulmonary tissues [5].

Accumulating data had reported that the histone deacetylase activity of sirtuin 1 (SIRT1) may play a crucial role as a protective mechanism against many disease states in which oxidative stress plays a key role [6]. Inhibition of SIRT1 expression was proven to have direct effects on the Keap1/Nrf2/ARE pathway, which, in turn, impedes antioxidant defense mechanisms and increases reactive oxygen species (ROS) production [7]. In the case of chemotherapy-induced pulmonary toxicity, these effects may subsequently increase the expression of transforming growth factor-beta 1 (TGF-β1), which has been thought to be the main trigger of the inflammatory and fibrogenic signals in the pulmonary tissues [8].

Recent studies have reported that the cross-talk between JAK-2 and STAT-3 signaling may play a fundamental role in the pathogenic events that may underlie the different forms of pulmonary toxicities induced by CPA [9]. Activation of the JAK2/STAT3 signaling pathway was proven to enhance fibroblast-to-mesenchymal transition, augment epithelial-to-mesenchymal transition, and modulate the pathways in which high mobility group box 1 (HMGB1) may be involved [10]. These events may proceed until reaching the induction of profound inflammatory and fibrotic reactions in the pulmonary tissues as a consequence of the administration of the chemotherapeutic agents [11].

Amentoflavone (AMV) is a flavonoid that had proven efficacy in the treatment of a wide range of disease states in which oxidative stress, inflammation, and apoptosis may play a pathophysiologic role [12,13,14]. These favorable effects might originate from the ability of AMV to affect ROS generation with subsequent modulation of the redox state and the inflammatory cascade in different body tissues [15]. In addition, the recent studies that have tried to explore the effect of AMV on autophagy/apoptosis balance exhibited promising results [16,17]. Therefore, the investigation of the potential effects of the different doses of AMV on CPA-induced pulmonary toxicity with the exploration of its antioxidant, anti-inflammatory, and apoptosis-modulating effects were the main objectives of the current research.

## 2. Materials and Methods

### 2.1. Chemicals and Drugs

CPA was obtained as a kind gift from Santa Cruz Biotechnology, Inc., Dallas, TX, USA (CAS No. 50-18-0). AMV and sodium carboxymethyl cellulose (CMC) were obtained in powder form from Sigma-Aldrich Co., St. Louis, MO, USA (CAS No. 1617-53-4 and 9004-32-4, respectively). All other chemicals and reagents employed were of analytical grade and were obtained from MyBioSource, San Diego, CA, USA. CPA was dissolved in distilled water. AMV was suspended in 0.5% sodium CMC solution.

### 2.2. Experimental Protocol

**Figure 1:**
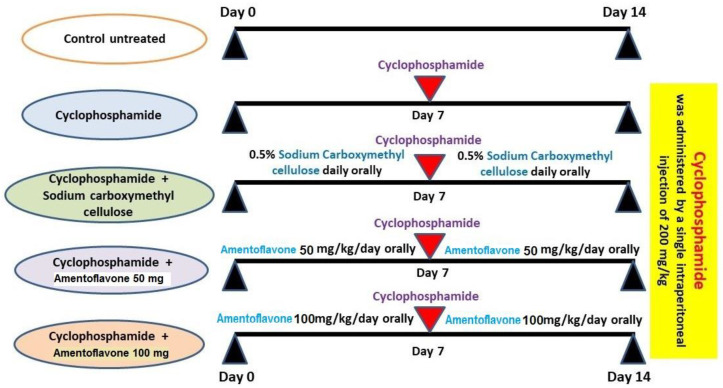
A schematic diagram for the schedule of animal treatment.

All the experiments in the present study were carried out following the U.K. Animals (Scientific Procedures-Act, 1986), EU Directive 2010/63/EU for animal experiments and were approved by the Standing Committee of Bioethics Research, Prince Sattam Bin Abdulaziz University, Saudi Arabia (approval number SCBR-004-2023). Forty male adult Wistar rats were allowed to acclimatize for two weeks with free access to food and water ad libitum with a 12:12 light/dark cycle. After that, these animals were randomly divided into five equal groups of eight rats per group, as demonstrated in Figure 1, as follows: control untreated group; CPA alone group; CPA group treated with 0.5% sodium carboxymethyl cellulose solution daily; CPA group treated with 50 mg/kg AMV daily [15]; and the CPA group treated with 100 mg/kg AMV daily [13]. A single dose of CPA (200 mg/kg) was injected intraperitoneally to induce pulmonary toxicity [4]. AMV and sodium carboxymethyl cellulose solutions were administered daily orally via the oral gavage technique starting one week before and continuing for one week after CPA injection.

At the end of the experiments, the animals were fasted overnight and then anesthetized using an intraperitoneal injection of thiopental sodium (40 mg/kg body weight). The trachea was cannulated and infused three times with 1 mL of sterile 0.9% sodium chloride solution for the collection of the bronchoalveolar lavage fluid (BALF). Thereafter, specimens from the right lung were washed with physiological saline, homogenized, and centrifuged at 1008× *g* for 15 min. The yielded supernatant was analyzed for biochemical parameters. In addition, specimens from the left lungs were prepared for further identification of the changes at the histopathological, immunohistochemical, and electron microscopic levels.

### 2.3. Quantification of the Changes in the Total and Differential Leucocytic Counts in BALF in the Studied Groups

BALF was centrifuged at 168× *g* for 15 min at 4 °C, and the resulting supernatant was used for the assessment of the biochemical parameters, as mentioned later. After that, the cell pellets were suspended in 1 mL of sterile saline, and a Hausser Scientific™ Nageotte Bright-Line™ hemocytometer (Thermo Fisher Scientific, Waltham, MA, USA) was employed for the quantification of the total and differential leucocytic counts according to Viall and LeVine [18].

### 2.4. Assessment of Lactate Dehydrogenase (LDH), Tumor Necrosis Factor-Alpha (TNF-α), Interleukin 15 (IL-15), and Monocyte Chemotactic Protein 1 (MCP-1) in BALF

An ELISA kit purchased from ABclonal, Woburn, MA, USA (catalog no. RK03786), was utilized for the assessment of LDH levels in BALF. Rat TNF-α level in BALF was assayed using an ELISA kit obtained from Elabscience, Houston, TX, 77079, USA (catalog no. E-EL-R2856). Both IL-15 and MCP-1 were quantified in BALF using ELISA kits supplied by Cusabio, Houston, TX, USA (code no. CSB-E07453r and CSB-E07429r, respectively). All assays of these parameters were carried out following the instructions of the provider’s guide.

### 2.5. Detection of the Levels of TGF-β1 and Nuclear Factor Kappa B (NF-κB) p65 in the Lung Tissues

The tissue levels of TGF-β1 and NF-κB (p65) in the lung specimens were assayed using ELISA kits supplied by Wuhan Fine Biotech Co., Ltd., Wuhan, Hubei, China (catalog no. ER1378 and ER1187, respectively), following the steps indicated by the vendor’s guide.

### 2.6. Assay of the Tissue Content of Malondialdehyde (MDA) and Total Antioxidant Capacity (TAC) in the Lung Specimens

The antioxidant status in the different studied groups was evaluated via the determination of the lung tissue content of MDA and TAC. An ELISA kit obtained from Quimigen S.L., Madrid, Spain (catalog no. MBS268427), was employed for the assessment of tissue MDA, whereas Sun Long, Hangzhou, Zhejiang, China, supplied the kit that was utilized for the determination of the lung tissue content of TAC. The assessment of these parameters was performed by following the instructions attached to the kits.

### 2.7. Quantification of Nuclear Factor (Erythroid-Derived 2)-like 2 (Nrf2), SIRT-1, and Kelch-like ECH-Associated Protein 1 (Keap1) Contents in the Lung Tissues

The lung tissue content of Nrf2 was quantified using an ELISA kit purchased from Novus Biologicals, Centennial, CO, USA (catalog no. NBP3-08161). Meanwhile, SIRT-1 levels in the pulmonary tissues were measured using kits supplied by Elabscience, Houston, TX, USA (catalog no. E-EL-R1102). MyBioSource, San Diego, CA, USA, was the provider of the ELISA kit used for the assessment of Keap1 levels in the pulmonary tissues (catalog no. MBS7218529).

### 2.8. Assessment of Janus Kinase 2 (JAK2) and Signal Transducer and Activator of Transcription 3 (STAT3) in the Pulmonary Tissues

The ELISA kit supplied by Abcam, Waltham, MA, USA (catalog no. ab253224), was utilized for the quantification of tissue JAK2 in the pulmonary tissues. Meanwhile, tissue STAT3 level was assayed in these specimens using the ELISA kit obtained from Wuhan Fine Biotech Co., Ltd., Wuhan, China (catalog no. ER0163), according to the vendor’s protocol.

### 2.9. Determination of Tissue Hydroxyproline and Matrix Metalloproteinase (MMP)-3 and -9 in the Pulmonary Tissues

Shanghai Coon Koon Biotech Co., Shanghai, China, was the provider of the ELISA kits used for the determination of hydroxyproline content of the pulmonary tissues (code number EA0040Ra). Following the manufacturer’s instructions, lung tissue MMP3 and MMP9 were measured using ELISA kits supplied by MyBioSource, San Diego, CA, USA (catalog no. MBS729026 and MBS722532, respectively).

### 2.10. Measurement of the Lung Tissue Content of Beclin 1 and LC3-II as Markers of Autophagy

The ELISA kit purchased from Abbexa Ltd., Cambridge, UK, was utilized for the assessment of beclin 1 levels in the pulmonary tissues (catalog no. abx256394). Quantification of the lung tissue levels of the autophagy-related protein LC3-II was implemented using the ELISA kit purchased from MyBioSource, San Diego, CA, USA (catalog no. MBS1600540), according to the vendor’s guide.

### 2.11. Analysis of the Histopathological Changes Induced by the Different Treatments in the Lung Tissue Specimens

Parts of the extracted lung tissue specimens were immersed in neutral-buffered formalin for fixation. Then, these specimens were dehydrated in ascending grades of alcohol, cleared using xylene, and embedded in paraffin wax. The resulting paraffin blocks were then cross-sectioned with a microtome at 5 µm thickness. These sections were deparaffinized, rehydrated, stained with hematoxylin and eosin, and examined under a light microscope (Olympus, Tokyo, Japan). The extent of pulmonary inflammation was assessed using the Szapiel scoring rules, where 0 denotes the absence of alveolar inflammation; 1 refers to mild alveolitis, which involves less than 20% of the lung tissue; 2 denotes moderate alveolar inflammation, which involves 20–50% of the lung tissue; and 3 refers to severe alveolar inflammation, which involves more than 50% of the lung tissue [11].

For the assessment of the fibrotic changes in the lungs, the deparaffinized sections were rehydrated, stained with Masson’s trichrome stain, and examined under a light microscope (Olympus, Tokyo, Japan). The degree of fibrosis was determined following the method outlined by Hubner et al. [19].

### 2.12. Detection and Quantification of the Immunoreactivity to Cleaved Caspase 3 Antibodies in the Pulmonary Tissues

The formalin-fixed, paraffin-embedded sections from the lung tissue were used for the assessment of the immunoexpression of cleaved caspase-3. Briefly, the lung tissue sections in which the cleaved caspase-3 antigen was unmasked were incubated with the primary rabbit monoclonal antibody for cleaved caspase-3 (ABclonal, Woburn, MA, USA, catalog no. A11021) overnight at 4 °C. After that, these sections were washed and then incubated with an anti-rabbit secondary antibody from MyBioSource, San Diego, CA, USA (catalog no. MBS9610384). Then, these sections were counterstained with hematoxylin, dehydrated using ascending grades of alcohol solution and xylene, and mounted with Permount mounting medium (ProSciTech, Thuringowa Central, QLD, 4817, Australia, code no. IA019). The slides were examined under a light microscope (Olympus, Tokyo, Japan) for the intensity of the positively stained cells and were graded as follows: mild (+), moderate (++), and strong (+++). Image J software (1.49 v) from the National Institutes of Health, the United States, was used for the determination of the percentage of cleaved caspase-3 immunoexpression in the pulmonary tissues [20].

### 2.13. Assessment of the Effect of CPA with or without AMV on the Electron Microscopic Picture of the Pulmonary Tissues

Parts of the left lobe of the lung were fixed in glutaraldehyde buffer solution and cut into small pieces of 1 mm thickness. Then, these sections were incubated in phosphate buffer for 24 h, after which they were fixed in a 1% osmium tetraoxide solution, dehydrated, and then embedded into resin. These specimens were then sectioned with an ultramicrotome (Leica EM UC7) at 1 µm thickness and stained with lead citrate and uranyl acetate. These sections were examined under a transmission electron microscope (Jeol Ltd., Tokyo, Japan) to detect the ultrastructural changes in the lung tissues.

### 2.14. Analysis of the Statistical Data

For statistical processing of the obtained data, GraphPad Prism Version 5.00 (San Diego, CA, USA) was utilized. The obtained results were expressed as the mean ± standard error of the mean (SEM). One-way analysis of variance (ANOVA) was used to compare the different studied groups, followed by the Tukey–Kramer post-hoc test. The probability level (*p*-value) of less than 0.05 was set as the minimum acceptable level to denote statistical significance.

## 3. Results

### 3.1. AMV Dose-Dependently Ameliorated the Effect of CPA Administration on the Total and Differential Leucocytic Counts in BALF

**Figure 2:**
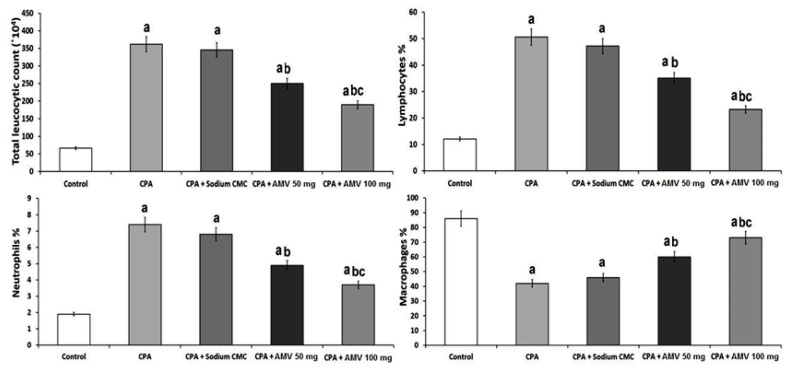
Effect of cyclophosphamide (CPA) with or without amentoflavone (AMV) on the total and differential leucocytic counts (mean ± SEM). ^a^ Significant compared vs. the control group; ^b^ significant compared vs. the CPA group; and ^c^ significant compared vs. the CPA + AMV 50 mg/kg group.

As demonstrated in Figure 2, CPA administration induced a significant elevation in the total leucocytic counts (TLCs) and the percentages of neutrophils and lymphocytes, associated with a significant reduction in the percentage of macrophages when compared versus the control group. Interestingly, AMV dose-dependently combatted the effects of CPA administration on the aforementioned parameters, with a significant reduction in the TLCs associated with a significant decrement in the percentages of lymphocytes and neutrophils and a significant increase in the percentage of macrophages.

### 3.2. AMV Administration, in a Dose-Dependent Manner, Abrogated the Perturbations in BALF LDH, TNF-α, IL-15, and MCP-1 Levels Induced by CPA Injection

**Figure 3:**
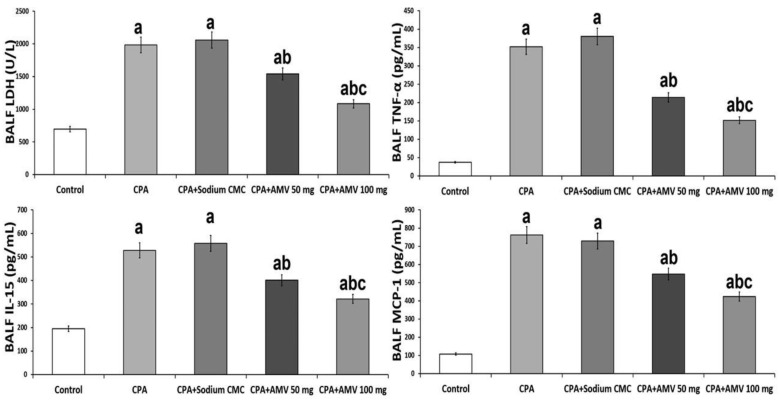
Effect of cyclophosphamide (CPA) with or without amentoflavone (AMV) on lactate dehydrogenase (LDH), tumor necrosis factor alpha (TNF-α), interleukin 15 (IL-15), and monocyte chemotactic protein 1 (MCP-1) levels in the bronchoalveolar lavage fluid (mean ± SEM). ^a^ Significant compared vs. the control group; ^b^ significant compared vs. the CPA group; and ^c^ significant compared vs. the CPA + AMV 50 mg/kg group.

As depicted in Figure 3, CPA administration elicited a significant elevation in the levels of LDH, TNF-α, IL-15, and MCP-1 in BALF when compared to the control group. AMV dose-dependently exhibited a unique ability to reduce these parameters to levels that approximate normal values.

### 3.3. AMV Dose-Dependently Reversed the Changes Elicited by CPA Injection in TGF-β1, NF-κB (p65), JAK2, and STAT3 Levels in the Pulmonary Tissues

**Figure 4:**
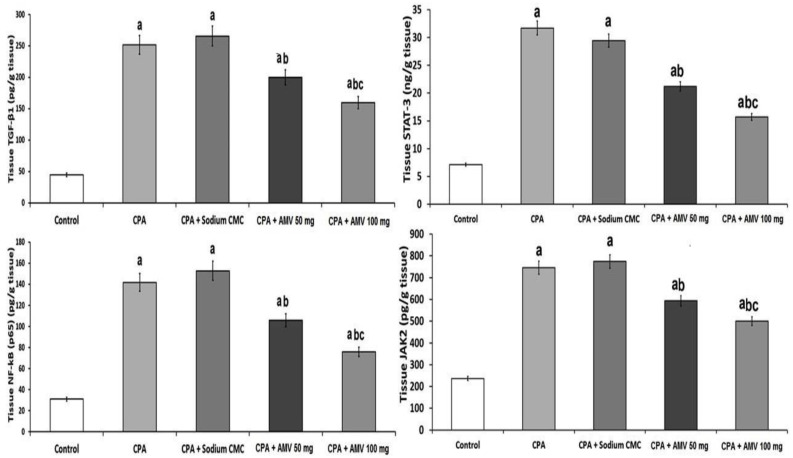
Effect of cyclophosphamide (CPA) with or without amentoflavone (AMV) on the levels of TGF-β1, NF-κB (p65), JAK2, and STAT3 in the pulmonary tissues (mean ± SEM). ^a^ Significant compared vs. the control group; ^b^ significant compared vs. the CPA group; and ^c^ significant compared vs. the CPA + AMV 50 mg/kg group.

Figure 4 demonstrates the effect of the different treatments on the tissue levels of TGF-β1, NF-κB (p65), JAK2, and STAT3. The significant elevation in the pulmonary tissue levels of these parameters created by CPA injection was ameliorated with the administration of AMV, and these changes were proven to be dose-dependent.

### 3.4. AMV Dose-Dependently Mitigated the Changes in MDA and TAC Levels in the Pulmonary Tissues Induced via CPA Injection

**Figure 5:**
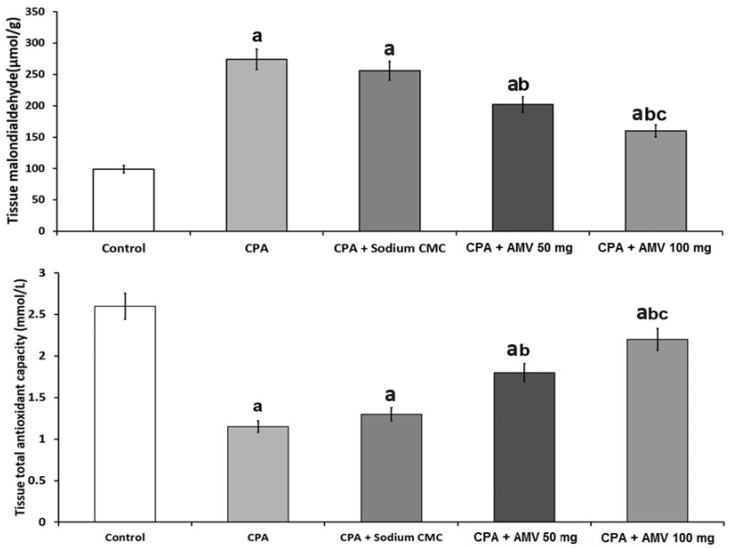
Effect of cyclophosphamide (CPA) with or without amentoflavone (AMV) on the levels of malondialdehyde (MDA) and the total antioxidant capacity (TAC) in the pulmonary tissues (mean ± SEM). ^a^ Significant compared vs. the control group; ^b^ significant compared vs. the CPA group; and ^c^ significant compared vs. the CPA + AMV 50 mg/kg group.

The role of oxidative stress in the pathogenesis of CPA-induced pulmonary toxicity is depicted in Figure 5. Significant decreases in tissue TAC and a significant elevation in tissue MDA level were demonstrated in rats treated with CPA alone. Interestingly, a significant dose-dependent decline in tissue MDA level associated with a significant dose-dependent elevation in tissue TAC level was noticed in CPA-injected rats treated with AMV relative to the group treated with CPA alone.

### 3.5. AMV Administration Impeded the Changes in Nrf2, SIRT-1, and Keap1 Levels in the Pulmonary Tissues Induced via CPA Injection

**Figure 6:**
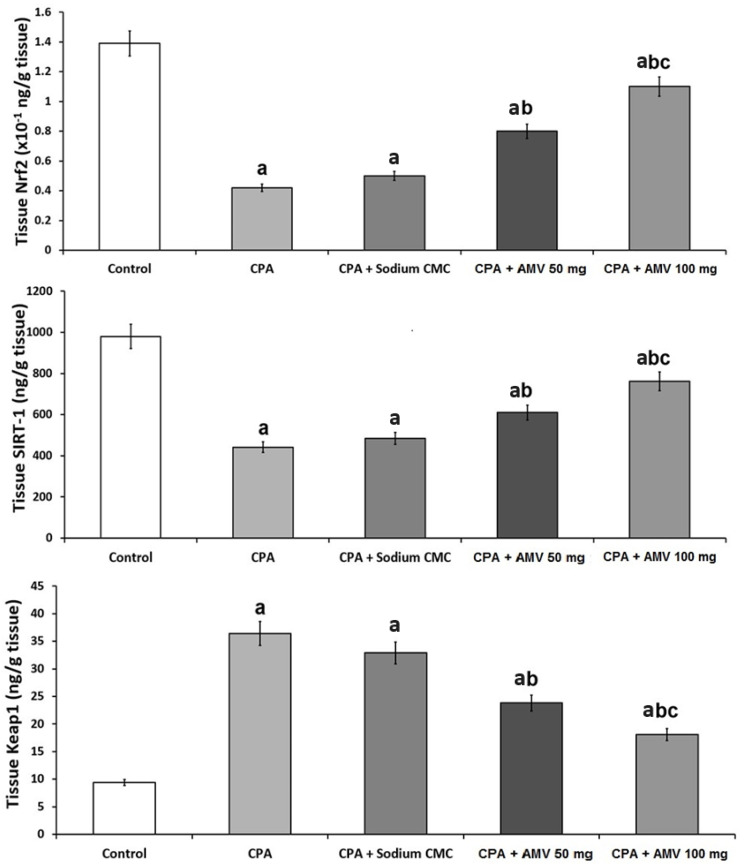
Effect of cyclophosphamide (CPA) with or without amentoflavone (AMV) on Nrf2, SIRT-1, and Keap1 levels in the pulmonary tissues (mean ± SEM). ^a^ Significant compared vs. the control group; ^b^ significant compared vs. the CPA group; and ^c^ significant compared vs. the CPA + AMV 50 mg/kg group.

As shown in Figure 6, rats treated with CPA alone exhibited a significant decline in tissue Nrf2 and SIRT-1 levels associated with a significant increase in tissue Keap1 levels when compared to the control group. AMV dose-dependently ameliorated these changes, with a significant elevation in the tissue levels of Nrf2 and SIRT-1 accompanied by a significant repression in tissue Keap1 levels when compared versus rats treated with CPA alone.

### 3.6. AMV Reversed the Changes Induced by CPA Injection in Hydroxyproline, MMP-3, and MMP-9 Levels in the Pulmonary Tissues

**Figure 7:**
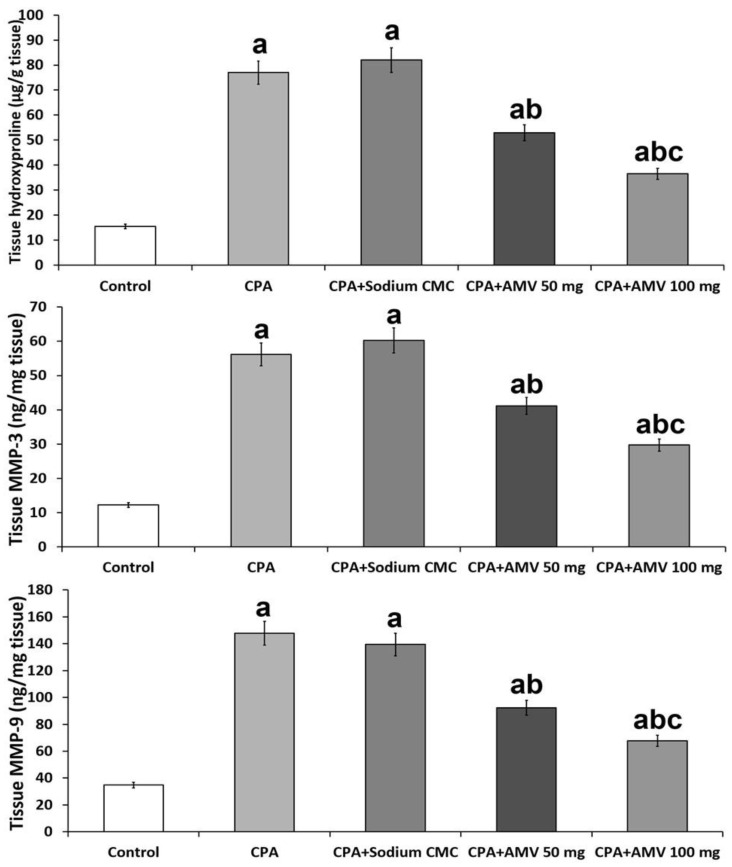
Effect of cyclophosphamide (CPA) with or without amentoflavone (AMV) on hydroxyproline, matrix metalloproteinase 3 (MMP-3), and MMP-9 levels in the pulmonary tissues (mean ± SEM). ^a^ Significant compared vs. the control group; ^b^ significant compared vs. the CPA group; and ^c^ significant compared vs. the CPA + AMV 50 mg/kg group.

Figure 7 shows the effect of the different treatments on lung tissue hydroxyproline, MMP-3, and MMP-9 levels. A significant increase in these parameters was reported in the CPA-treated animals relative to the control group. A significant dose-dependent decrease in the levels of hydroxyproline, MMP-3, and MMP-9 in the pulmonary tissues was noticed in the CPA-injected animals treated with AMV relative to the rats that received CPA alone.

### 3.7. Effect of the Different Treatments on the Markers of Autophagy

**Figure 8:**
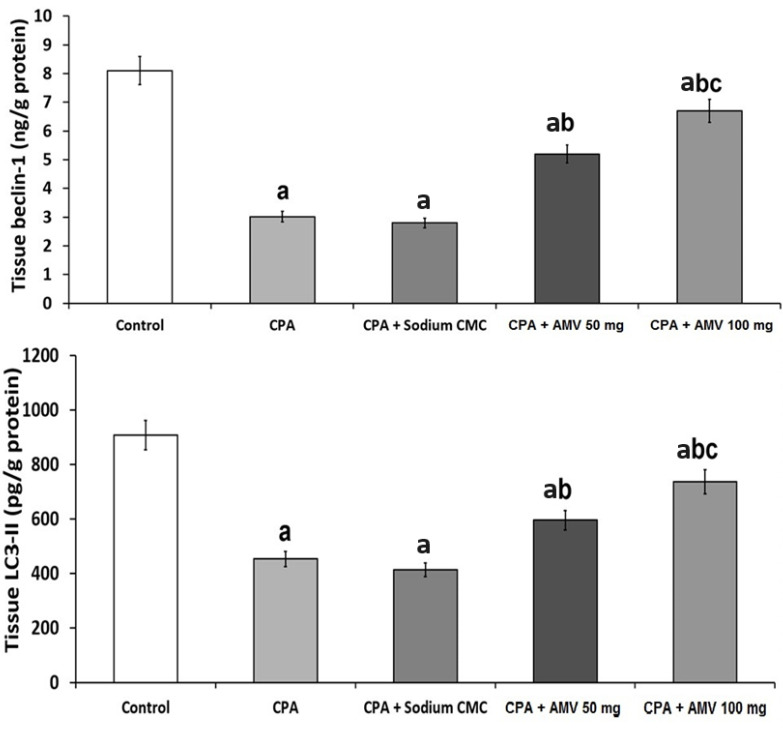
Effect of cyclophosphamide (CPA) with or without amentoflavone (AMV) on the markers of autophagy (mean ± SEM). ^a^ Significant compared vs. the control group; ^b^ significant compared vs. the CPA group; and ^c^ significant compared vs. the CPA + AMV 50 mg/kg group.

The role of autophagy in the pathogenesis of CPA-induced pulmonary toxicity is depicted in Figure 8. The significant diminution in the levels of beclin 1 and LC3-II in the pulmonary tissues created by CPA injection was abrogated with the administration of AMV in a dose-dependent manner.

### 3.8. Effect of the Administration of the Different Doses of AMV on the Histopathological Changes in the Pulmonary Tissues Induced via CPA Injection

**Figure 9:**
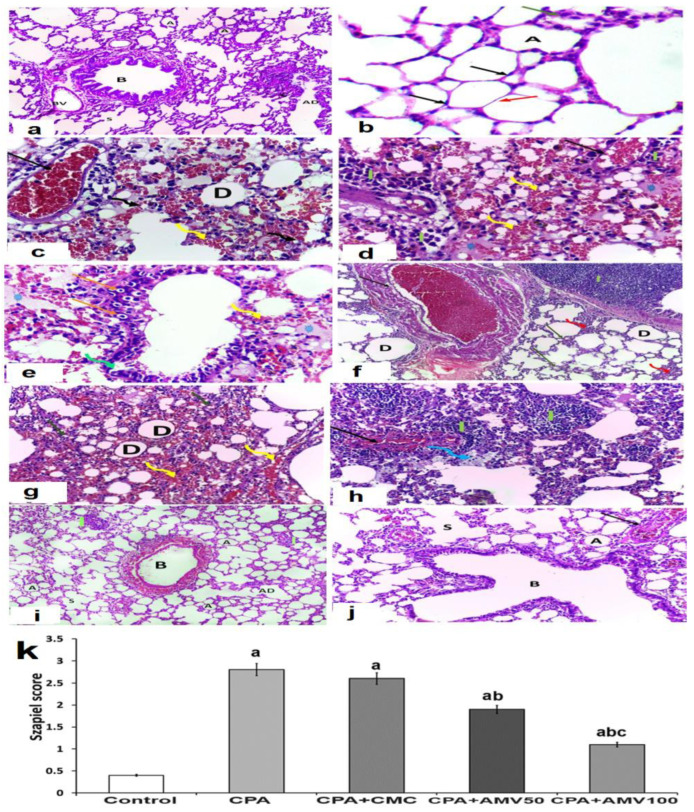
Representative microscopic images of sections of the lung stained with hematoxylin and eosin from (**a**) the control group showing normal lung architecture with a clear alveolar duct (AD), alveolar sacs (S), alveoli (A), blood vessels (BV), and bronchioles with clear lumen (B) (×200); (**b**) the control group with a thin interalveolar septum (red arrow) with flattened type I pneumocytes (black arrows) and cuboidal type II pneumocytes with rounded nuclei (green arrow) lining the alveolar space (A) (×1000); (**c**,**d**) cyclophosphamide group showing massive interstitial hemorrhage (yellow arrows), diffuse alveolar collapse (curved black arrow), and other dilated alveoli (D), along with heavy inflammatory cellular infiltration (I) with massive vascular congestion (black arrow) (×400); (**e**,**f**) cyclophosphamide group treated with sodium CMC showing severe congestion of the lung vasculature (black arrow) and interstitial hemorrhage (yellow arrow), severe inflammatory cellular infiltration (I), distorted bronchiolar walls (green arrow), necrosis of bronchial epithelium with pyknotic nuclei and perinuclear vacuolation (orange arrow), thick interalveolar septa (red arrow), and diffuse eosinophilic exudate (astrix) (e: ×400 and f: ×200); (**g**,**h**) cyclophosphamide group treated with 50 mg/kg amentoflavone daily showing still-dilated alveoli (D) with significant amounts of inflammatory cellular infiltration (I, green arrow) and vascular congestion (black arrow) with interstitial hemorrhage (yellow arrow) and presence of foamy macrophages (blue arrow) (×200); (**i**,**j**) cyclophosphamide group treated with 100 mg/kg amentoflavone daily showing apparently normal alveoli (A), alveolar sacs (s), alveolar ducts (AD), and folded bronchioles (B) with minimal inflammatory cellular infiltration (I) and minimal vascular congestion (black arrow) ((i): ×100 and (j): ×200); and (**k**) Szapiel scores of the different studied groups. ^a^ Significant compared vs. the control group; ^b^ significant compared vs. the cyclophosphamide (CPA) group; and ^c^ significant compared vs. the CPA + amentoflavone (AMV) 50 mg/kg group.

Sections from the lung tissues of the control group showed that the lung parenchyma appeared to consist of alveolar ducts, alveolar sacs, and different-sized clear alveoli in between which thin septa were noticed. Different-sized bronchioles that had intact folded mucosa with a regularly arranged smooth muscle layer and blood vessels with normal thickness were observed (Figure 9a). The alveoli showed a single lining layer of two cell types, one of which was type I pneumocytes (thin squamous cells with flat nuclei). Type II pneumocytes appeared cuboidal with rounded nuclei (Figure 9b). Sections from the animals injected with CPA alone showed a massive destruction of the alveolar spaces with heavy infiltration of the interstitial tissues with inflammatory cells, and severe vascular congestion (Figure 9c,d). The administration ofsodium CMC solution did not exert any significant effects on the histopathological changes induced by CPA injection (Figure 9e,f). These changes were ameliorated with the administration of AMV, which produced a dose-dependent decrease in the infiltration of the pulmonary tissues, with inflammatory cells associated with a significant mitigation of the vascular congestion and restoration of the normal architecture of the alveoli and the interalveolar septa. These admirable effects mediated by AMV were associated with a dose-dependent decrease in the fibrotic changes in the pulmonary tissues relative to the group treated with CPA alone (Figure 9g–k).

### 3.9. Effect of the Administration of the Different Doses of AMV on the Amount of Collagen Fibers Deposited in the Pulmonary Tissues Induced by CPA Injection

**Figure 10:**
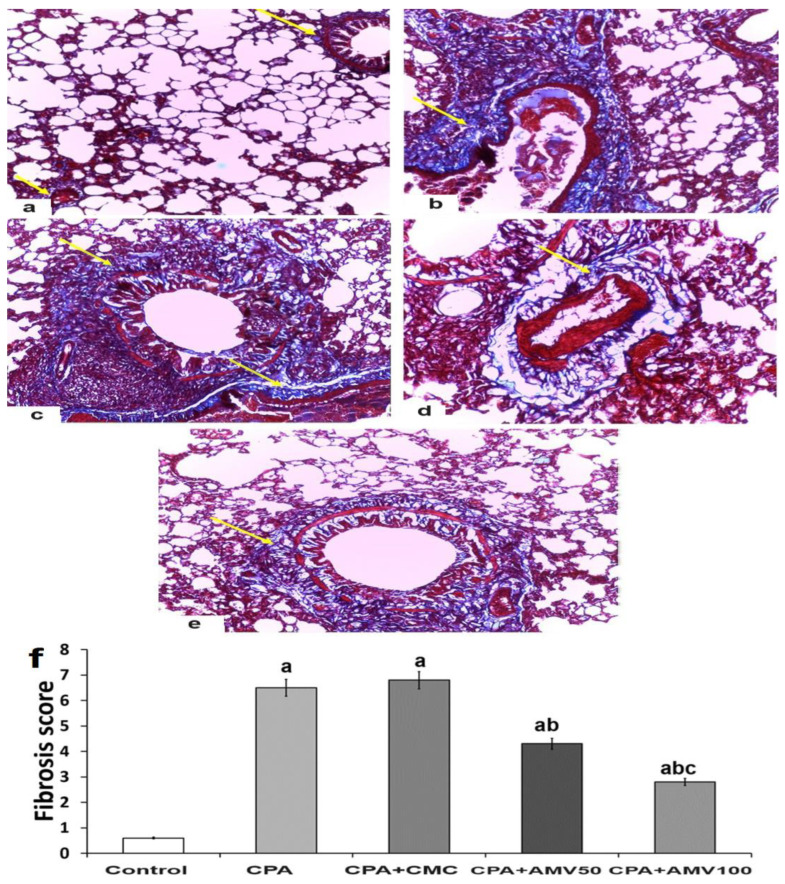
Representative microscopic images of sections of the lung stained with Masson’s trichrome stain from (**a**) the control group show a minimal amount of collagen fibers around the bronchioles and the blood vessels (yellow arrows); (**b**) the cyclophosphamide (CPA) group; and (**c**) the CPA group treated with sodium CMC showing a massive deposition of collagen fibers around the bronchioles, the blood vessels, and within the interalveolar septa (yellow arrows); (**d**) the CPA group treated with 50 mg/kg amentoflavone (AMV) daily; (**e**) the CPA group treated with 100 mg/kg AMV daily, showing diminution in the amount of the collagen fibers around the alveoli, the bronchioles, and the pulmonary vasculature (yellow arrows); and (**f**) fibrosis scores of the different studied groups (^a^ significant compared vs. the control group; ^b^ significant compared vs. the CPA group; and ^c^ significant compared vs. the CPA + AMV 50 mg/kg group).

Masson’s trichrome stain revealed that there was little collagen fiber deposition in the inter-alveolar septa, walls of blood vessels, and around bronchioles in the control group (Figure 10a,f). Collagen fibers of the lungs of the CPA group and the CPA group treated with sodium CMC showed a marked deposition in the interstitium, the walls of blood vessels, and the walls of bronchioles (Figure 10b,c,f). Treatment with AMV showed a dose-dependent amelioration in the amount of collagen fibers deposited in the interalveolar septa, walls of the bronchi, and around the walls of the blood vessels (Figure 10d–f).

### 3.10. Effect of the Different Doses of AMV on the Changes in the Immunoexpression of Cleaved Caspase 3 in the Pulmonary Tissues of Rats Injected with CPA

**Figure 11:**
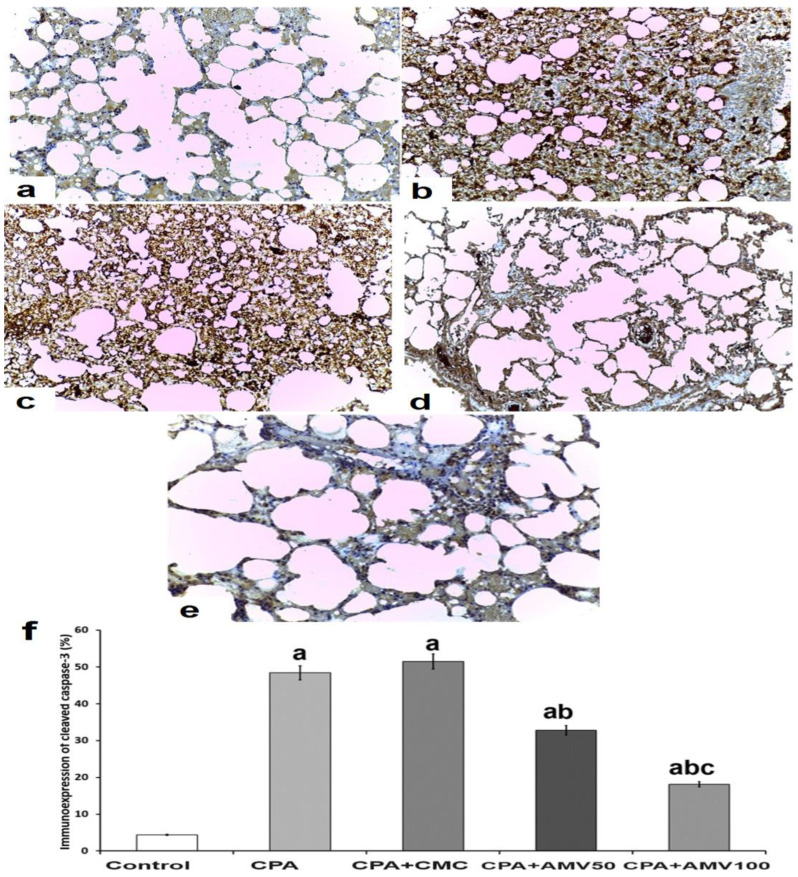
Representative microscopic images of lung sections of immunohistochemical staining of cleaved caspase-3 (×200) from (**a**) the control group showing minimal positive staining for cleaved caspase-3; (**b**) the CPA group; and the (**c**) CPA group treated with sodium CMC exhibiting strong positive staining for cleaved caspase-3; (**d**) the CPA group treated with 50 mg/kg AMV daily with moderately positive staining for cleaved caspase-3; (**e**) the CPA group treated with 100 mg/kg AMV daily revealing mildly positive staining for cleaved caspase-3; and (**f**) percentages of the immunoexpression of cleaved caspase-3 (^a^ significant compared vs. the control group; ^b^ significant compared vs. the CPA group; and ^c^ significant compared vs. the CPA + AMV (AMV) 50 mg/kg group).

Animals injected with CPA showed a strong, positive immunoexpression of cleaved caspase 3 compared to that expressed by the control group (Figure 11a,b,f). Although the administration ofsodium CMC solution failed to affect these changes elicited by CPA injection (Figure 11c,f), the administration of AMV dose-dependently reduced the immunoexpression of cleaved caspase 3 in the lung tissues relative to the animals treated with CPA alone (Figure 11d–f).

### 3.11. AMV Combatted the Effects of CPA Injection on the Electron Microscopic Picture of the Specimens Extracted from the Pulmonary Tissues

**Figure 12:**
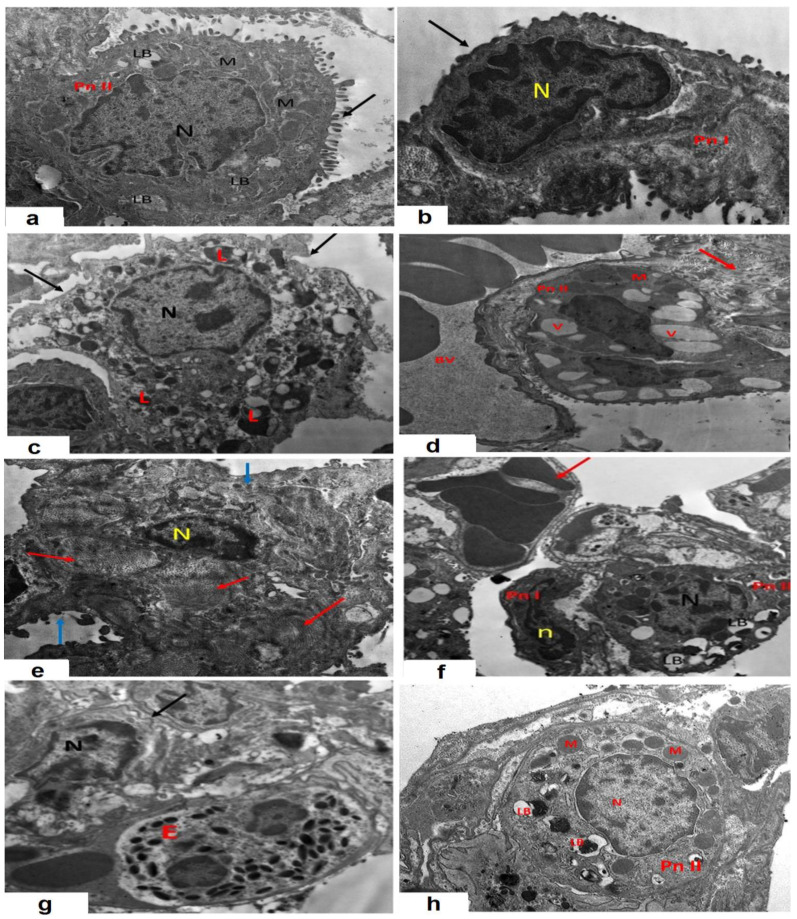
Representative transmission electron microscopic images of ultra-thin sections of the lung from (**a**) the control group showing type II pneumocytes with a prominent microvillous border (black arrow) and euchromatic nuclei (N). These cells exhibit lamellar bodies (LB) and mitochondria (M) in their apical cytoplasm; (**b**) the control group showing type I pneumocytes with a flat nucleus (N) and small rim of cytoplasm; (**c**) the control group showing alveolar macrophages with an indented nucleus (N), irregular outlines (black arrow), and multiple cytoplasmic lysosomes (L); (**d**) the cyclophosphamide (CPA) group showing type II pneumocytes with vacuolated lamellar bodies (V) together with disrupted mitochondria (M), deposited collagen within the inter-alveolar septum (red arrow), and congested blood vessels (BV); (**e**) the CPA group showing macrophages with a kidney-shaped nucleus (N) within the inter-alveolar septum (blue arrow) with collagen deposition (red arrow); (**f**) the CPA group treated with sodium CMC showing type II pneumocytes with a rounded nucleus (N) with marginal chromatin clumping, marked cytoplasmic vacuolation, and degenerated lamellar bodies (LB); (**g**) the CPA group treated with 50 mg/kg AMV daily showing marked narrowing of the alveolar space with infiltration of eosinophils (E) and macrophages with a kidney-shaped nucleus (N) with irregular outlines (arrow); and (**h**) the CPA group treated with 100 mg/kg AMV daily demonstrated that type II pneumocytes regained most of their characters, with a prominent microvillus border and euchromatic nuclei (N) with lamellar bodies (LB), and collagen was still observed deposited in the inter-alveolar septum.

The administration of CPA elicited significant changes in the electron microscopic picture when compared to that of the control group (Figure 12a–e). This was evidenced by the appearance of numerous vacuolated lamellar bodies and many cytoplasmic vacuoles in type II pneumocytes, which showed short, irregular microvilli. The nuclei were dark and irregular, with marginal chromatin clumping. These changes induced by CPA were not significantly ameliorated with the administration of sodium CMC (Figure 12f). Interestingly, AMV dose-dependently ameliorated these changes with the regaining of the microvillous border of type II pneumocytes, with a significant reduction in the numbers of cytoplasmic vacuoles and degenerated lamellar bodies, and the restoration of the nuclear structure to an apparently normal picture (Figure 12g,h).

## 4. Discussion

The induction of pulmonary toxicity represents an important drawback of the use of CPA in clinical practice [21]. Up till now, the underlying etiological factors involved in the pathogenic pathways of these toxic reactions have not yet been fully explored, despite reports that mitigation of the antioxidant defense mechanisms, induction of aggressive inflammatory cellular responses, enhancement of the expression of the profibrotic agents, and impaction of the balance between the apoptotic and autophagic signals were implicated in the pathogenesis of CPA-induced pulmonary toxicity [22]. Therefore, recent research has been directed towards the exploration of the effect of targeting these pathways using non-traditional agents in an attempt to combat the pulmonary toxicities elicited by CPA injection [4].

In the current study, the administration of CPA induced a significant increase in BALF TLC, which was obviously manifested as a significant elevation in the percentages of BALF neutrophils and lymphocytes, associated with a significant diminution in the percentage of BALF macrophages relative to the control group. These events were in the same line with previous reports that emphasized on the role of inflammation in the pathogenesis of the pulmonary toxicities induced by CPA administration [23]. Hassanein et al. [24] reported that treatment with CPA was associated with massive inflammatory cellular infiltration of the pulmonary tissues, mainly with neutrophils and lymphocytes, which reflects the role of the inflammatory processes in this pathologic condition. The significant decrease in the percentage of BALF macrophages in the present study may be attributed to the inhibitory effect of the pro-inflammatory cytokines on the pathways of the activation of the macrophages [25]. This deprives the pulmonary tissues from the anti-inflammatory and antiapoptotic effects of the activated macrophages, thereby aggravating CPA-induced pulmonary toxicity [26]. These events were dose-dependently reversed in the current work with the administration of AMV, which reveals its role as an effective anti-inflammatory agent [27].

TGF-β1 is considered a key mediator involved in the pathogenesis of CPA-induced pulmonary toxicity [28]. Activation of epithelial-to-mesenchymal transition mediated by TGF-β1 may be attributed to the affection of smad3 signaling with subsequent activation of the fibroblastic foci in the lungs [29]. In addition, TGF-β1 was proven to be an important regulator of MMP-3 and MMP-9 expression in the pulmonary tissues, which efficiently participate in the pathogenesis of lung fibrosis induced by chemotherapeutic agents [30]. Moreover, the interplay between TGF-β1 and NF-κB expression, with subsequent affection of the inflammatory pathways in which the NLRP3 inflammasome plays a key role, might be an additional mechanism that may illustrate the toxic effects of CPA administration on the lungs [31]. The present study confirmed these data, as evidenced by the significant increase in the pulmonary tissue levels of TGF-β1, LDH, TNF-α, IL-15, and MCP-1 associated with the significant elevation in the tissue levels of NF-κB, MMP-3, and MMP-9 induced by CPA administration relative to the control group. Interestingly, the significant decrease in tissue TGF-β1 levels was reported in the current work with the administration of the different doses of AMV, which was directly reflected in the tissue levels of the pro-inflammatory cytokines and fibrogenic mediators with the net result of the amelioration of the fibrogenesis and inflammatory processes [15].

Accumulating evidence has shed light on the effect of targeting JAK-2/STAT-3 signaling on the pathogenesis of CPA-induced pulmonary toxicity [32]. As a consequence of CPA administration, the growth factors that are overexpressed in the lung, such as fibroblast growth factor, platelet-derived growth factor, and TGF-β1, as well as the profibrotic/pro-inflammatory cytokines, were reported to augment the activity of the JAK2/STAT3 axis, which initiates cellular changes that may be incriminated in the pulmonary toxicities induced by CPA [33]. These changes include the induction of fibroblast-to-mesenchymal transition, enhanced epithelial-to-mesenchymal transition, modulation of autophagy and apoptosis, induction of cell cycle arrest, and alteration of the protein folding capacity of the endoplasmic reticulum [34]. In addition, activation of the JAK2/STAT3 axis was proven to increase the genetic expression of high mobility group box 1 (HMGB1), which has an intimate relationship with the inflammatory and fibrotic changes that occur in the lung microenvironment in response to the lung injury induced by the chemotherapeutic agents [35]. This role was confirmed in the current study, where targeting JAK2/STAT3 signaling using the different doses of AMV was able to significantly ameliorate the different aspects of pulmonary toxicities induced by CPA administration. These results were in line with Rabidas et al. [17], who attributed the inhibitory properties of flavonoids, such as AMV, on the JAK2/STAT3 axis to its ability to modulate toll-like receptor 4 (TLR4)/NLRP3 inflammasome signaling.

SIRT1 belongs to the sirtuin family of proteins that have histone deacetylase activity, which plays a crucial role in cell differentiation and metabolism [36]. The affection of SIRT1 expression was suggested as a possible mechanism of CPA-induced pulmonary toxicity [37]. This may originate from the fact that SIRT1 has a unique ability to modulate the Keap1/Nrf2/ARE pathway, possibly via mitigating Keap1 expression, which, in turn, enhances the transcriptional activity of Nrf2 and augments ARE-binding ability [38]. This may be reflected in the increased expression and activity of heme oxygenase 1, with subsequent inhibition of ROS overproduction and alleviation of the deleterious effects of oxidative stress in the pulmonary tissues [39]. In addition, SIRT1 was proven to modulate the expression of TGF-β1 in the pulmonary tissues with a subsequent decremental decrease in the fibrogenic mediators [40]. Moreover, SIRT1 was reported to combat the inflammatory processes in the pulmonary tissues through the inhibition of NF-κB-regulated gene expression, possibly via the deacetylation of the p65 subunit of NF-κB at lysine residues [41]. This was confirmed by the results of the present study, where the significantly decreased levels of SIRT1 in the pulmonary tissues in rats treated with CPA alone were associated with a significant increase in Keap1 levels, decreased Nrf2 content, and a significant decrement of the antioxidant defense mechanisms in the pulmonary tissues relative to the control group. Interestingly, these characteristic effects of SIRT1 inhibition by CPA were dose-dependently mitigated in the present work with the administration of AMV. These favorable effects may be attributed to the ability of AMV to increase the genetic expression of SIRT1 with a subsequent increase in Nrf2 content in the pulmonary tissues, which restores the pro-oxidant/antioxidant balance to normal values [16,42]. In addition, the enhancement of the histone deacetylase activity of SIRT1 mediated by AMV may underlie the mitigating effects of AMV detected in the present study on the inflammatory cascade [16,43].

Recent reports had revealed that MMPs play an important role in the pathogenesis of the pulmonary toxicities elicited by CPA [44]. Administration of CPA was reported to increase the production of TGF-β1, which, in turn, modulates the activities of MMP-3 and MMP-9 in the pulmonary tissues [45]. This consequently leads to extracellular matrix remodeling and modulation of the expression of the pro-inflammatory cytokines TNF-α and IL-1β, thereby affecting the inflammatory process in the pulmonary tissues [46]. As revealed in the current study, the increased expression of tissue MMP-3 and MMP-9 in the lungs of CPA-treated animals was associated with a significant increase in the lung tissue content of hydroxyproline, which highlighted the role of MMP-3 and MMP-9 in the fibrogenic events induced by CPA [47]. Interestingly, AMV in the present study had the ability to produce a dose-dependent decrease in the pulmonary tissue levels of hydroxyproline, MMP-3, and MMP-9, which signifies its role as a potent antifibrotic agent, possibly derived from its modulatory effects on TGF-β1 expression [48].

Autophagy is an important intracellular process that plays a crucial role in the maintenance of cellular homeostasis and the regulation of cellular differentiation and survival [49]. The affection of the mediators of autophagy was regarded as a possible explanation for the toxic effects of CPA on the lungs [5]. As evidenced in the current study, the autophagic stream was proven to be significantly inhibited in the lung tissues following the administration of CPA. The significant decrease in the number of autophagosomes, the defective fusion between the autophagosomes and the lysosomes, and elevated intracellular levels of p62 are among the characteristic features that signify the inhibitory effects of CPA on the autophagic functions in the lungs [26]. These findings were thought to occur as a consequence of the cross-talk between the different signaling pathways, including Akt/MAPK/mTOR signaling, the JAK2/STAT3 pathway, and the Keap1/Nrf2 axis [4,28,50]. Modulation of the autophagic stream in the lungs was reported to induce extracellular matrix deposition, respiratory epithelial cell dysfunction, induction of myofibroblast transformation, upregulation of the TGF-β1 signaling pathways, induction of massive apoptosis of the fibroblasts, and initiation of the cellular events related to epithelial-to-mesenchymal transition [51]. Interestingly, AMV in the present study exhibited a unique dose-dependent ability to combat the deleterious effects of CPA on autophagic functions. This effect may be explained by the modulatory effects of AMV on the key regulatory molecular pathways of autophagy, including Keap1/Nrf2, JAK2/STAT3, and Akt/MAPK/mTOR signaling pathways [13,17].

Recent studies have paid attention towards the potential role played by apoptosis in the pathogenesis of pulmonary toxicities induced by CPA administration [4]. The increased production of ROS, together with increased expression of the pro-inflammatory cytokines elicited by CPA administration, was reported to enhance apoptosis on both the extrinsic and intrinsic levels, with the net result of significant damage to the pulmonary tissues [52]. In addition, the significant decrement in the autophagic stream elicited by CPA injection was reported to be an important trigger of fibroblast apoptosis [53]. Moreover, the inhibition of the genetic expression of the antiapoptotic proteins is an additional mechanism that may explain the deleterious effects of CPA on the pulmonary tissues [26]. Interestingly, AMV in the present study revealed an outstanding ability to combat the apoptosis-inducing effects of CPA administration on the pulmonary tissues via decreased tissue levels of cleaved caspase-3. This was in agreement with Li et al. [12], who attributed the major part of the desirable effects of AMV on cellular viability and survival to its ability to enhance the genetic expression levels of the antiapoptotic molecules in addition to its unique mitigating effects on the activities of the key apoptotic mediators, including caspase 1, caspase 3, and caspase 9, with subsequent restoration of the histopathological picture to levels that approximate the normal values.

The limitations of the present work include the relatively small number of animals and the short duration of the study. Further research involving large animal groups for extended periods of time is recommended to allow for the assessment of the long-term toxic effects of CPA on the pulmonary tissues. In addition, further research is required to measure the extent of inflammation and pulmonary fibrosis. Moreover, there is an inevitable need to assess whether the pulmonary toxicities encountered in this animal model are reversible or irreversible; this is because the pulmonary toxicities encountered in patients treated with CPA seem to be irreversible. Another important limitation was that the present study did not determine whether AMV affects the antitumor activity of CPA or not. Furthermore, the present study did not assess the potential safety of AMV for patients treated with CPA.

## 5. Conclusions

**Figure 13:**
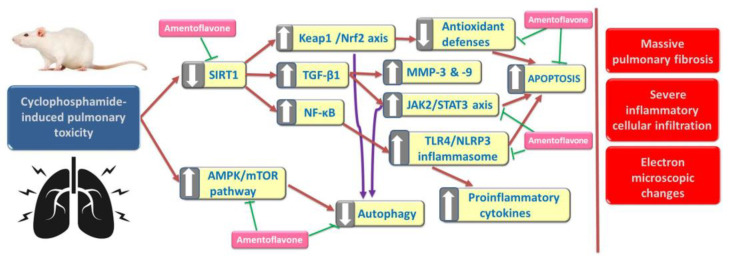
A schematic diagram of the possible mechanisms by which amentoflavone (AMV) may combat the toxic effects of cyclophosphamide (CPA) on the lungs.

The administration of AMV may open new horizons towards the amelioration of the toxic effects on the lungs induced by CPA. This conclusion may rely on the promising effects of AMV administration on several signaling pathways involved in the pathogenesis of this condition, including modulation of the SIRT-1/Nrf2/Keap1 axis, mitigation of the inflammatory and fibrotic events mediated via TGF-β1/NF-κB signaling, affection of the JAK-2/STAT-3 axis, decreased expression of MMPs, and modulation of the markers of autophagy and apoptosis in the lung tissues (Figure 13). Future research is recommended for longer time periods to assess the long-term effects of CPA on the lungs, and to determine the exact molecular mechanisms by which AMV may ameliorate these effects. In addition, future studies should evaluate the safety of AMV and its potential effects on the antitumor activities of CPA. Furthermore, future experiments should evaluate the possibility of applying the findings of the present study in clinical settings.

## Data Availability

Data are available from the corresponding author upon reasonable request.
